# In Vivo Assessment on Freeze-Cast Calcium Phosphate-Based Scaffolds with a Selective Cell/Tissue Ingrowth

**DOI:** 10.1021/acsami.4c12715

**Published:** 2024-10-21

**Authors:** Lucie Pejchalová, Jaroslav Pejchal, Jakub Roleček, Michaela Vojníková, Zdeněk Chlup, Vojtěch Mařák, Manuela González-Sánchez, Jana Čížková, David Salamon

**Affiliations:** † Central European Institute of Technology, Brno University of Technology, Purkynova 656/123, 612 00 Brno, Czech Republic; ‡ Department of Toxicology and Military Pharmacy Faculty of Military Health Science, University of Defence, Trebesska 1575, 500 01 Hradec Kralove, Czech Republic; § Department of Chemistry, Biochemistry Mendel University in Brno, Trida Generala Piky 1999/5, 613 00 Brno, Czech Republic; ∥ Institute of Physics of Materials, Academy of Science of the Czech Republic, Zizkova 513/22, 616 62 Brno, Czech Republic; ⊥ Department of Physics of Condensed Matter, Faculty of Physics, University of Seville, Avenue de la Reina Mercedes, S/N, Seville 41012, Spain; # Department of Radiobiology, Faculty of Military Health Science, University of Defence, Trebesska 1575, 500 01 Hradec Kralove, Czech Republic; ¶ Institute of Structural and Functional Ceramics, Montatuniversität Leoben, Peter Tunner Strasse 5, 8700 Leoben, Austria

**Keywords:** in vivo, tissue engineering, scaffolds, calcium phosphates, freeze-casting, bioceramics

## Abstract

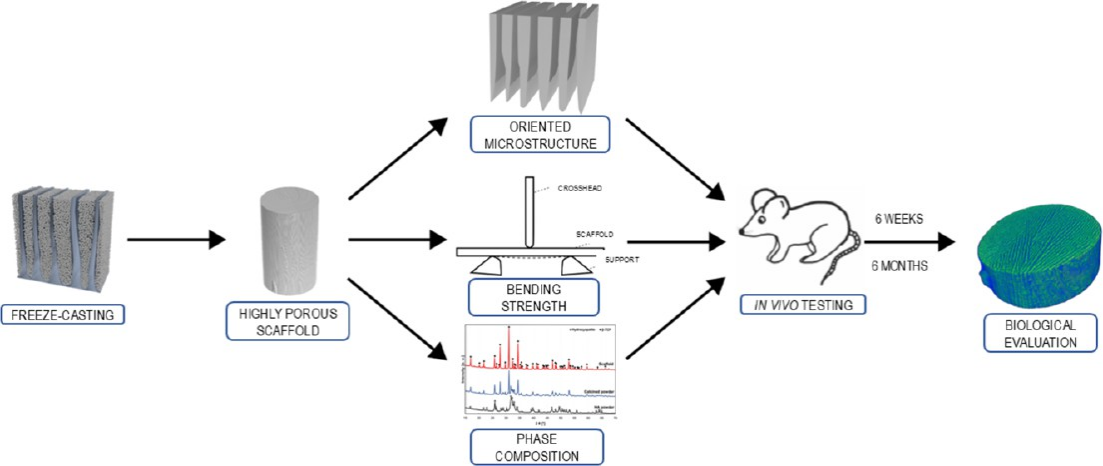

Highly porous bioceramic scaffolds are widely used as bone substitutes in many applications. However, the use of bioceramics is often limited to hard tissues due to the risk of potential soft tissue calcification. A further limitation of highly porous bioceramic scaffolds is their poor mechanical stability, manifested by their tendency to break under stress. In our study, highly porous CaP-based scaffolds were prepared via freeze-casting with longitudinal and oriented pores ranging from 10 to 20 μm and a relative porosity of ∼70%. The resulting scaffolds achieved a flexural strength of 10.6 ± 2.7 MPa, which, in conjunction with their favorable bioactivity, made them suitable for in vivo testing. The prepared scaffolds were subcutaneously implanted in rats for two distinct periods: 6 weeks and 6 months, respectively. The subsequent development of fibrous tissue and involvement of myofibroblasts, newly formed vessels, and macrophages were observed, with notable changes in spatial and temporal distributions within the implantation. The absence of calcification in the surrounding soft tissue, as a result of the narrow pore geometry, indicates the opportunity to tailor the scaffold behavior for soft tissue regeneration.

## 1 Introduction

Tissue engineering is still evolving as a part of regenerative medicine. Since the 1950s, the focus of research has transitioned from tissue replacement to enhancing tissue regeneration and assisted self-repair. The third generation of bioceramics or bioceramic-organic composites remains a significant research focus.^1^ Calcium phosphate (CaP)-based bioceramics, such as hydroxyapatite (HA), α- and β-tricalcium phosphates (TCP), and their doped or modified alternatives, represent a promising option for regenerative medicine because of the chemical and phase composition close to the human hard tissues.^2,3^ Biocompatibility, bioactivity, and potential biodegradation are the main benefits of CaPs. In the context of bioceramics, biocompatibility can be explained as the ability of a material to be in contact with living tissue without inducing an adverse immune reaction. Herein, bioactivity is defined as the ability of the material to bond with the surrounding tissue actively. Both factors are crucial, but other factors also affect successful regeneration, including the topography, surface roughness, specific surface area, total porosity, pore size, and mechanical performance of scaffolds.^2−4^ Although bioceramics can be utilized as porous scaffolds, they cannot usually meet all of the requirements. Scaffold processing usually provides the opportunity to tailor and control total porosity and pore size and shape, improving vascularization and transfer of metabolites after implantation but at the expense of mechanical stability. The scaffold must always be strong enough to withstand the implantation process and recovery period while maintaining its structural integrity. The mechanical performance of CaP-based bioceramic scaffolds is typically constrained by their inherent low strength and brittleness; therefore, they require reinforcement or shaping techniques in a special way.^1,4,5^

Freeze-casting appears to be a promising shaping method for bioceramics or other biomaterials because it allows the preparation of highly porous scaffolds with biomimetic microstructure and sufficient mechanical stability (for bioceramics in order of MPa).^5−7^ The internal microstructure (pore size and shape) depends on several parameters, including solvent type, solid loading of suspension, and freezing conditions.^8,9^ Bioceramic scaffolds with elongated pores can be obtained by unidirectional freezing of a water-based ceramic suspension.^8,10^ The final microstructure of freeze-cast scaffolds is typically anisotropic, with open porosity consisting of ceramic walls (lamellae) and pores (interlamellar spaces) between them. The mechanical stability of freeze-cast bioceramic scaffolds is usually improved by the presence of interlamellar bridges, resulting from the use of freezing additives such as sucrose, poly(vinyl) alcohol (PVA), gelatin, etc.^11^ The primary mechanism by which bioceramics gain their strength is sintering, which helps to reinforce the scaffold’s microstructure as the ceramic particles sinter together. However, in the case of CaP bioceramics, thermal treatment, such as sintering, can also induce the phase transformation: HA (>900 °C) → β-TCP (>1200 °C) → α-TCP. This phase transformation may change the biodegradation rate and result in the loss of mechanical performance in the long term perspective,^2,3^ which could lead to the scaffold’s failure. Thus, scaffolds with a medium degradation rate (β-TCP) are often the most suitable because they offer controlled support for newly forming tissue for an appropriate period of time.^12^

Several studies have reported the suitability of CaP-based bioceramics for bone regeneration. By contrast, only a few studies have focused on using bioceramics to regenerate other kinds of soft tissue. This is mainly due to the high risk of calcification (stimulation of stem cells to differentiate into osteoblasts) of the soft tissue surrounding the scaffold. The risk of calcification of soft tissue is particularly high when the tissue regeneration takes a long time and the material degrades or dissolves while releasing Ca^2+^ and PO_4_^3–^ ions.^2,13,14^

The selection of the pore size and shape may also act as a limiting factor for the ingrowth of specific cell types. Open macropores with a size between 100 and 300 μm (or higher) promote angiogenesis and vascularization and naturally attract bone cells (osteoblasts).^4,14^ However, there is a possibility that small open pores (10–30 μm) will selectively induce the ingrowth of cells with smaller size (fibroblasts, neurons, and skin cells) while still allowing adequate angiogenesis. Larger cells, such as osteoblasts, will not simply enter the microstructure of the scaffold in the early stages after implantation. However, with the advancing material degradation and dissolution, the Ca^2+^ and PO_4_^3–^ ions will become accessible for osteoblasts, thus facilitating the process of hard tissue formation in the vicinity. Wieringa et al.^15^ have reviewed a similar approach for polymeric (collagenous) scaffolds for neural guidance, mentioning freeze-cast scaffolds with lamellar microstructure as an alternative to autografts and other scaffolds used for neural tissue regeneration.

Our study aims to prepare highly porous CaP bioceramic scaffolds with small-sized directional pores (limiting osteoblasts) via freeze-casting and to characterize their physical and chemical properties. The suitability of the scaffolds for soft tissue regeneration is assessed in vivo. The biocompatibility, soft tissue ingrowth, and short- and long-term reactions of living organisms, including the absence of soft tissue calcification or ectopic bone formation, with proposed scaffolds are evaluated.

## 2 Materials and Methods

### 2.1 Bioceramic Powder Processing

HA powder (purum p.a., >90%, Sigma-Aldrich, USA) was calcined at 800 °C for 1 h in an air atmosphere to remove residual calcium-based compounds and form a biphasic mixture of HA and β-TCP.

### 2.2 Bioceramic Suspension Preparation

The calcined biphasic powder was used to prepare the freeze-casting suspension. Ceramic powder (15 vol %) was dispersed in a water-based solution. PVA (Mowiol 10–98, Sigma-Aldrich, USA) was used as an organic binder (1.3 vol %, according to the whole volume). Several additives, such as dispersant (Dolapix CE 64; Zschimmer & Schwarz, Germany), sucrose (3 wt %, according to solid loading), and octanol (purum, 98%, Honeywell Fluka, Germany), were used to stabilize the bioceramic suspension and modify freezing behavior. Once the suspension had been prepared, it was placed on an in-house-made roller mixer (∼190 rpm) for at least 24 h to ensure the mixture was adequately homogenized.

### 2.3 Freeze-Casting and Sintering

The freeze-casting method was used to prepare porous CaP scaffolds. Generally, the freeze-casting process requires a mold with a thermally conductive bottom, which ensures heat transfer between the suspension and the freezing medium. In this work, a 3D-printed mold from poly(lactic) acid (PLA) (Plasty Mladec, Czech Republic) was used with an inner diameter of 25 mm, a height of 45 mm, and a copper block embedded in the bottom. To obtain samples with a size suitable for in vivo testing, the 3D-printed mold was filled with plastic tubes with a diameter of approximately 3 mm. The mold was initially precooled to −5 °C and filled with a degassed suspension. Subsequently, liquid nitrogen was poured around the copper block of the mold, ensuring fast cooling and a high freezing rate. Once the suspension was completely frozen, samples were extracted from the plastic tubes, and the freeze-drying process was commenced. The samples were freeze-dried in an in-house-made lyophilization apparatus for 24 h at a pressure of ∼10 Pa, with the temperature gradually rising to ∼40 °C to sublime ice crystals that had formed during freezing without damaging the lamellar structure. After freeze-drying, cylindrical green bodies (thin and long rods) with a lamellar microstructure were obtained. In the final stage of the process, the green bodies were conventionally sintered at 1200 °C for 2 h in an air atmosphere using a muffle furnace (CLASIC CZ, Czech Republic).

### 2.4 Phase Analysis

Raman spectra of the as-received powder, biphasic powder mixture, and final scaffold were acquired using a WITec Alpha 300R (WITec, Germany) with a 532 nm wavelength laser operating at 40 mW.

The as-received powder, biphasic powder mixture, and sintered scaffolds were further investigated by X-ray diffraction (XRD) analysis using an X-ray diffractometer (Rigaku, Japan) equipped with a Cu Kα anode as a radiation source. The measurement was performed at a voltage of 40 kV and a current of 30 mA in the Bragg–Brentano mode. Acquired XRD spectra were compared with referential data to identify the present phases. Additionally, the content of HA and β-TCP was determined by the simple height law for *I*_100_ peaks. The percentage of HA was calculated by the following formula^16^
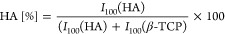


### 2.5 Microscopical Observations

The lamellar microstructure, intergranular porosity, and tissue ingrowth after in vivo testing were investigated by high-resolution scanning electron microscopy (SEM) Verios 460L (FEI, Czech Republic) and LYRA3 (Tescan, Czech Republic). Before the microscopical observation, samples were coated (EM ACE600, Leica, Germany) with 18 nm of carbon to enhance the micrograph quality.

Images of interlamellar spaces were acquired by a digital stereomicroscope (Motic, Hong Kong). The size of interlamellar spaces (pore size) was determined by a measuring tool in the ImageJ software. The specific surface area of the sintered scaffold was determined by nitrogen adsorption with the Brunauer–Emmett–Teller (BET) method (Micro300C, 3P Instruments, Germany).

Tissue ingrowth, changes in microstructures, and degradation of implanted scaffolds were observed by X-ray computed microtomography (CT) with a Zeiss Versa Xradia 610 device (Zeiss Group, Germany). The scanning was performed at a voltage of 50 kV and a current of 89.9 μA, with a voxel size of 3.4038 μm. ImageJ and AVIZO software were used for image processing.

### 2.6 Mechanical Properties

Flexural strength was determined for a minimum of 30 sintered samples (with a length of approximately 20 mm) via the three-point bending test using a universal testing machine (ZwickRoell Z50, ZwickRoell, Czech Republic) to allow Weibull statistical analysis. The cross-head speed of 0.1 mm/min and a 16 mm span were used to load round bars with a diameter of 2 mm and a length of 20 mm.

### 2.7 Bioactivity Assessment

The bioactivity of sintered CaP scaffolds was tested in a simulated body fluid (SBF) solution prepared according to Kokubo and Takadama.^17^ Before soaking, the scaffolds were dried and weighed using the analytical balances XPS 204 (Mettler Toledo, USA) to evaluate the weight change. Scaffolds were cultivated using the laboratory orbital incubator/shaker ES-20 (Biosan, Latvia) at 37 °C in 2.5 mL of SBF for 21 days. Subsequently, the scaffolds were washed with deionized water and dried at room temperature. Then, the scaffolds were weighed again, and the bioactivity was assessed by the presence of precipitated HA, which was additionally confirmed by SEM.

### 2.8 Animal Experiment

#### 2.8.1 Animals

Female Wistar rats (aged 12–16 weeks and weighing 214–264 g) were purchased from Velaz (Czech Republic). The rats were randomly assigned into four groups, each consisting of 6 animals. The animals were kept in an air-conditioned room (22 ± 2 °C, 50 ± 10% relative humidity, with light from 7:00–19:00 h) in the accredited facility (accreditation no. 4284/2021-MZE-18134) and were allowed access to standard food and tap water ad libitum. The experiment was approved by the Ethics Committee of the Faculty of Military Health Sciences (approval no. MO 302220/2021-1457, Hradec Kralove, Czech Republic). All in vivo studies were performed by following ethical guidelines.

#### 2.8.2 Sample Sterilization

Before implantation, all CaP scaffolds were sterilized using hot air sterilization HS 121 A (Chirana, Czech Republic) at 170 °C for 40 min.

#### 2.8.3 Experimental Design

The rats were divided into short-term implantation (6 weeks) and long-term implantation (6 months) groups.

After an acclimatization period, the animals were anesthetized with a mixture of one volume of xylazine (Rometar 20 mg/mL) and 19 volumes of ketamine (Narkamon 100 mg/mL; both from Bioveta, Czech Republic). This solution was administered intramuscularly at 0.5 mL/kg of body weight. Under anesthesia, the rats were fastened onto an underlay and shaved on the back from the neck to the lumbar spine. The operation site was disinfected using a povidone-iodine solution (Betadine, EGIS, Czech Republic). Following the principles of the sterile technique, a 1 cm transversal incision was made between the shoulder blades. Two 20 mm deep pouches were formed subcutaneously in the caudal and cranial directions. One sterilized CaP scaffold (ca. Ø2 × 10 mm) was carefully placed into each pouch. The incision was then sutured using a sterile atraumatic, nonabsorbable suture (Chirmax, Czech Republic). Both control groups underwent the same procedure, except for the CaP scaffold implantation. After the procedure, the animals were checked daily for any signs of discomfort or infection. The sutures were removed 14 days after the surgery. Subsequently, the rats were observed at least three times per week.

#### 2.8.4 Sample Harvest

The samples were collected 6 weeks and 6 months after the surgical procedure. The animals were euthanized at each interval using an isoflurane (Piramal Healthcare, India) vapor overdose.

Venous blood was collected into heparinized tubes (Scanlab Systems, Czech Republic), and a volume of 120 μL was evaluated using the ABX Pentra 60C analyzer (Trigon-Plus, Czech Republic) to measure peripheral blood cell counts. All samples were measured three times, and the resulting values were averaged.

Subcutaneous samples were first macroscopically checked. The cranial piece was carefully removed from its fibrous capsule and placed in 70% ethanol (Penta, Czech Republic) to fix and stabilize the scaffolds with tissue. Scaffolds with surrounding tissue for SEM were dehydrated by water extraction into ethanol at an increasing concentration. Drying in the drying chamber followed to keep the scaffolds as dry as possible. The extraction of water prevented the vaporization of water molecules in the microscope. For histopathological analysis, the CaP caudal scaffolds were carefully incised with the surrounding tissue and fixed with 10% neutral buffered formalin (Bamed, Czech Republic).

#### 2.8.5 Histopathological Sample Processing

After the fixation, the CaP samples were demineralized using a DC1 decalcifier (Bamed, Czech Republic). The samples were then dehydrated through increasing concentrations of ethanol and xylene and transferred into liquid paraffin (all from Bamed, Czech Republic) using a Leica TP1020 tissue processor (Leica, Germany). Five 5 μm thick sections were cut (Microtome saw model SM2000 R, Leica, Germany) from each paraffin-embedded sample and rehydrated (xylene and decreasing ethanol concentrations). Samples were stained with hematoxylin and eosin (both from Merck, USA) or aniline blue Masson Trichrome kit according to the manufactureŕs instructions (cat. no. 010210; DiaPath, Italy) or utilized for immunohistochemistry. The stained samples were then dehydrated and mounted in a nonaqueous dibutyl phthalate polystyrene xylene mounting medium (Merck, USA).

#### 2.8.6 Immunohistochemical Detection

Immunohistochemical detection of alpha-smooth muscle actin (αSMA, a marker of smooth muscles and myofibroblasts), platelet endothelial cell adhesion molecule (also known as CD31, a marker of endothelial cells), and CD68 (a marker of tissue macrophages) was performed using a standard peroxidase technique according to the procedure published previously.^18^ Rabbit recombinant monoclonal *anti*-αSMA antibody (AB150301, 1:50), rabbit recombinant multiclonal anti-CD31 antibody (AB281583, 1:50; all from Abcam Limited, UK), or rabbit recombinant multiclonal anti-CD68 antibody (AB303565, 1:50) were used as primary antibodies, while horseradish peroxidase-conjugated goat antirabbit antibody (AB205718, 1:2000; Abcam Limited) was utilized as the secondary antibody. Finally, 0.05% 3,3′-diaminobenzidine tetrahydrochloride chromogen solution in phosphate-buffered saline containing 0.02% hydrogen peroxide (all from Sigma-Aldrich) was added to visualize the antigen–antibody complex in situ.

#### 2.8.7 Histopathological Sample Evaluation

Stained samples were observed using a BX-51 microscope (Olympus, Japan). A basic histopathological assessment was performed in hematoxylin-eosin-stained samples.

The extent of fibrous tissue ingrowth (aniline blue Masson Trichrome-stained samples) and expression of αSMA (*anti*-αSMA-stained samples) were evaluated according to the semiquantitative score as 0 (none), 1 (1–25%), 2 (26–50%), 3 (51–75%), 4 (76–99%), and 5 (complete). Because the collagen fibers and *anti*-αSMA positive cells were not expressed homogeneously, the parameter represents an average of four values obtained from four quadrants measured from the outer fiber capsule to the center of the sample. Finally, an average with a standard deviation was calculated for the whole group.

Maximum vessel ingrowth was assessed in Masson Trichrome-stained and anti-CD31-stained samples. Four microphotographs were taken at 200-fold original magnification using a DP73 camera (Olympus, Japan), each randomly from one quadrant. The microphotographs were then divided into four sections. In each section, a vessel-like structure most distant from the fibrous capsule toward the center of the sample was further specified by combining the microphotograph and the microscope (at 600-fold original magnification). Finally, the shortest distance between the vessel-like structure and the fibrous capsule was measured using the ImagePro 5.1 computer image analysis system (Media Cybernetics, USA). Each sample represents an average of 16 measured values.

Finally, the expression of CD68 was assessed at 200-fold magnification in the microscopic field oriented to the scaffold tissue near the fibrous capsule interface and scored according to the semiquantitative score as 0 (baseline, ≤40 CD68 positive cells per microscopic field), 1 (mildly increased, 41–80 CD68 positive cells per microscopic field), 2 (moderately increased, 81–120 CD68 positive cells per microscopic field), or 3 (highly increased, >120 CD68 positive cells per microscopic field).

## 3 Results and Discussion

### 3.1 Freeze-Casting and Microstructure

**Figure 1:**
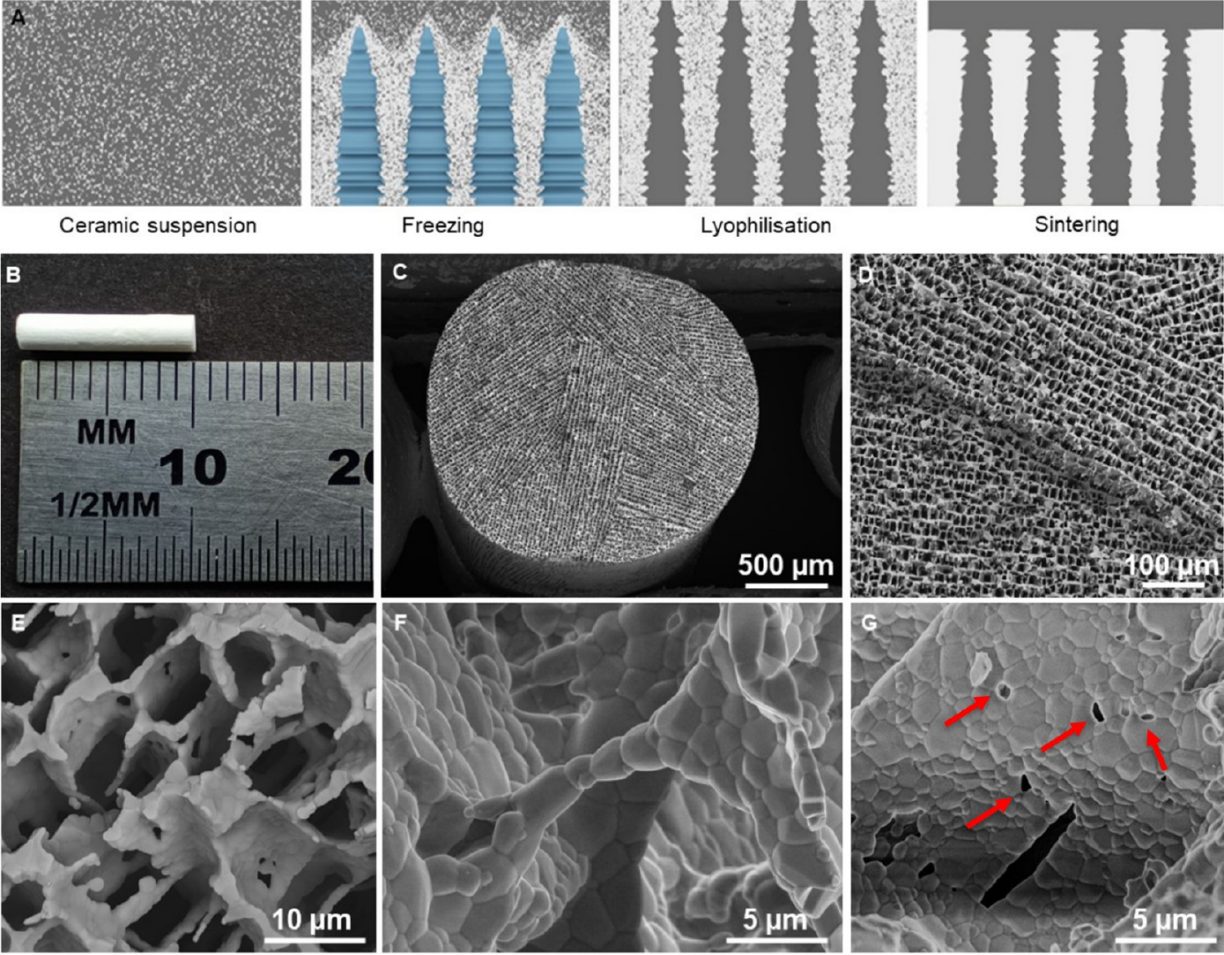
Detailed microstructural analysis of prepared scaffolds; (A) schematic illustration of the freeze-casting process; (B) scaffold before implantation; (C) oriented lamellar structure; (D) detail of lamellar structure; (E) interlamellar bridges; (F) detail of interlamellar bridge; and (G) intergranular pores (marked with red arrows).

The biomimetic microstructure of prepared scaffolds achieved via freeze-casting (Figure 1A) has promising applications in soft tissue engineering. Prepared freeze-cast scaffolds for in vivo testing had a diameter of approximately 2 mm and a length of 10 mm (Figure 1B). Scaffolds exhibited an oriented lamellar microstructure. The processing parameters of freeze-casting (freezing front velocity, used solvent, and additives) provided longitudinal and oriented pores (interlamellar spaces) with pore sizes ranging from 10 to 20 μm (Figure 1C,D). The microstructure was anisotropic and fully open, with pore size sufficient for soft tissue ingrowth and vascularization.^15,19^ However, it can be considered insufficient for bone cells.^4^ Ectopic bone formation was observed within the pores with a size of more than 100 μm (ideally around 300 μm).^4,12,20^ The proposed scaffolds have been designed for soft tissue regeneration, and the prepared pore size seems to be suitable for this purpose. Wieringa et al.^15^ summarized that having a pore size between 5 and 30 μm limits the ingrowth of unwanted tissue but still allows the diffusion of nutrients and elimination of metabolic products. Polymeric freeze-cast scaffolds were proposed as suitable because they could offer safe and directive tracks for growing neurons (through interlamellar spaces with sizes from 10 to 20 μm, ideally). This provides a potential application for the proposed bioceramic scaffolds. Since bioceramics dissolve slower than polymers, bioceramic scaffolds can offer protection and directed pathways for extended healing periods. Furthermore, the bioceramics preserve their shape in the liquid environment (they do not expand), so the pore size is also stable.

We also observed other microstructural features such as dendrites, interlamellar bridges (Figure 1E,F), and intergranular pores on lamellae (Figure 1G, marked with red arrows). Dendrites and interlamellar bridges facilitate the attachment of ingrowing tissue and cell adhesion,^21^ while intergranular pores ensure the better transfer of nutrients, metabolites, and signaling molecules throughout the scaffold.^12^ The open porosity surpassed 70%, which resulted from the low solid loading of the freeze-cast suspension. Previous studies demonstrated that porosity is necessary for maximized tissue ingrowth and can provide spaces for extracellular matrix formation. This fact was strongly reflected in the tested scaffolds, where even the chosen pore size (10–20 μm) was not limiting for tissue ingrowth. Another limiting factor can be a specific surface area of 1.53 g/m^2^. Some studies have shown that the low value of a specific surface area may affect the degradation behavior and rate and limit potential cell adhesion.^12,22,23^ However, we did not observe any limitation in tissue adhesion in our scaffolds, mainly due to the open porosity and interconnected pores.

### 3.2 Phase Analysis

Raman spectroscopy and XRD analysis revealed changes in the phase composition of the used powders as a cause of applied thermal treatment. Phase transformation of used CaP, which occurred during the scaffold preparation, was more than desirable.

**Figure 2:**
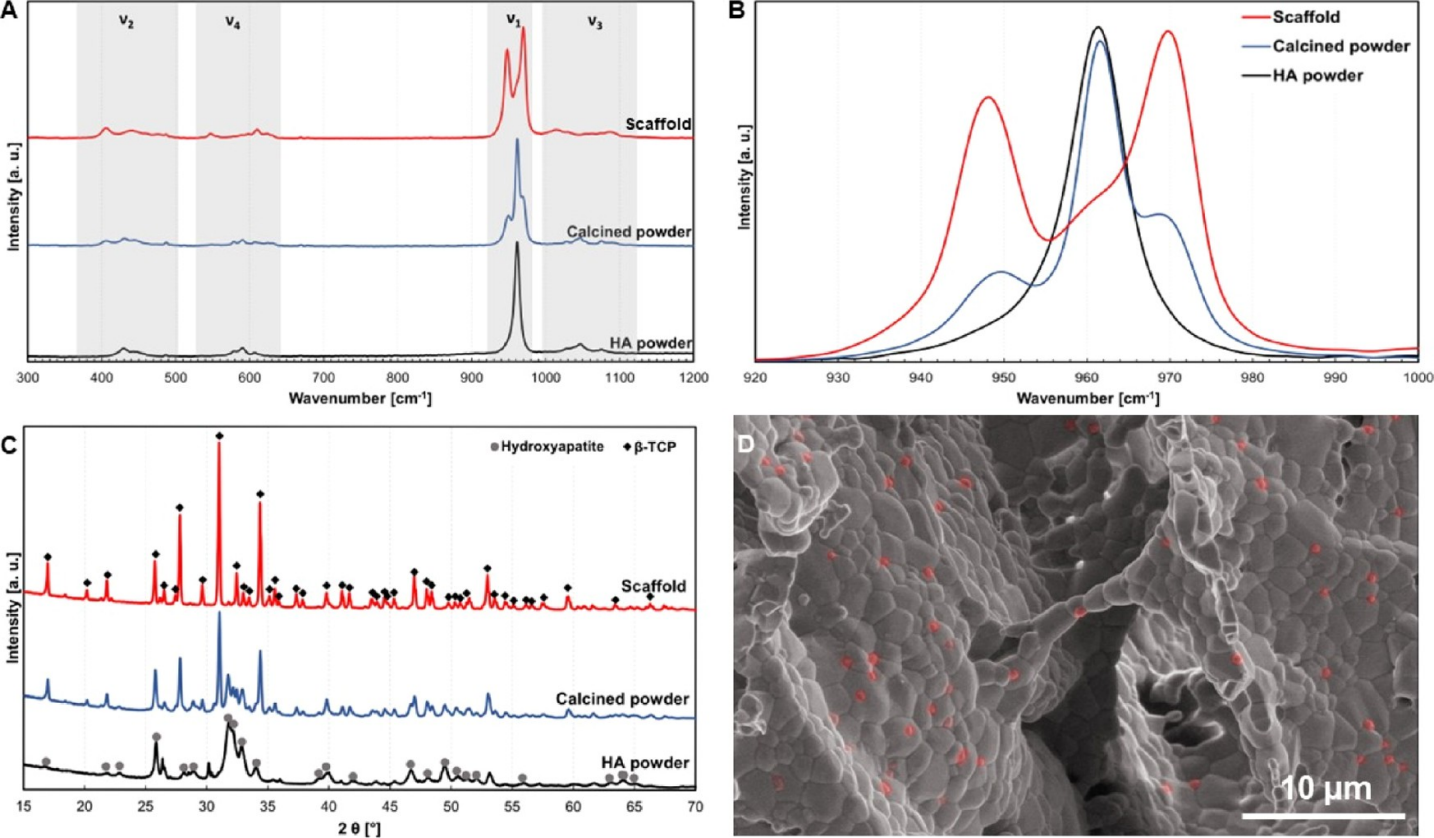
Phase analysis of CaPs; (A) comparison of Raman spectra; (B) Raman spectra of ν_1_ vibrational mode; (C) comparison of XRD spectra; and (D) SEM micrograph of β-TCP and HA grains (highlighted with red color).

Raman spectra of the as-received HA powder, biphasic powder mixture, and sintered scaffold are shown in Figure 2A. All spectra exhibited a typical vibrational spectrum of CaPs dominated by the internal PO_4_^3–^ modes. These are high-intensity ν_1_ (920–980 cm^–1^), accompanied by ν_2_ (370–505 cm^–1^), ν_3_ (995–1120 cm^–1^), and ν_4_ (530–645 cm^–1^). A closer look at the ν_1_ mode allows distinguishing between HA and β-TCP phases (Figure 2B). While the as-received HA powder spectrum contained only a single peak at 961 cm^–1^ (black line), the sintered scaffold showed two high-intensity peaks at 948 and 970 cm^–1^ and a shoulder at ∼960 cm^–1^ (red line). The calcined powder spectrum combined both features and contained three apparent peaks at 949, 961, and 970 cm^–1^ (blue line). Another typical feature of the HA Raman spectrum is the relative proximity of the ν_2_ and ν_4_ modes, which was observed for the as-received HA powder. In contrast, the modes in the β-TCP spectrum are further apart, as seen in the scaffold spectrum.^24,25^ However, due to the limited resolution of the instrument, no other residual phases could be directly detected.

A deeper analysis by XRD revealed that the as-received powder primarily contained HA with residual Ca-based compounds (Figure 2C, dark gray line). As HA is one of the most stable CaPs, it typically does not degrade or dissolve in the physiological environment, which indicates it may not be suitable for application in soft tissue.^2^ Therefore, the calcination (800 °C/1 h) applied before scaffold preparation helped to partially transform HA into the biphasic mixture of β-TCP and HA in a ratio of 70:30 (Figure 2C, blue line). As the powder calcination was set to 800 °C, the phase transformation remained incomplete.^26,27^ Following heat treatment (sintering) at 1200 °C for 2 h induced further phase transformation, resulting in the biphasic mixture of β-TCP and HA (Figure 2C, red line), but with a higher amount of β-TCP (∼95%). A low amount of HA remaining in the mixture was probably caused by decreased solid-state diffusion in freeze-cast scaffolds, which is the main driving force behind CaP sintering and phase transformation.^28^ However, in our case, considering that only approximately 5% of the HA remained, the effect on the degradation and dissolution of the scaffolds should be minimal.

The presence of residual HA was proved by SEM, where a low amount of homogeneously dispersed small grains was observed (Figure 2D, highlighted by red color). The issue of residual HA can potentially be resolved by raising the sintering temperature to achieve a complete HA → β-TCP phase transformation. Nevertheless, it is crucial to acknowledge that a sintering temperature exceeding 1300 °C may result in the phase transformation of β-TCP to α-TCP, loss of mechanical characteristics, and a higher degradation rate.^2,3,29,30^

### 3.3 Mechanical Performance

**Figure 3:**
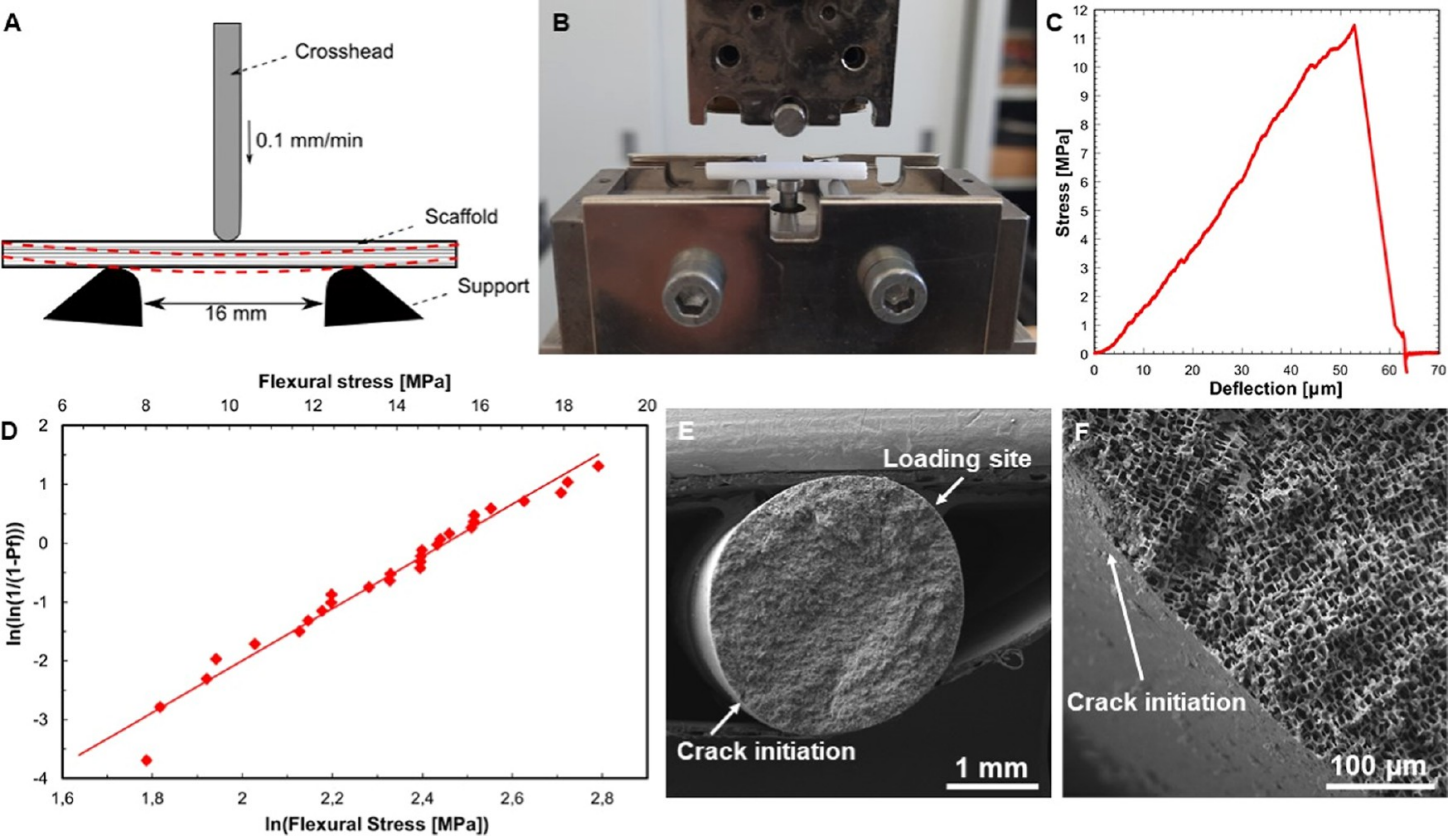
Mechanical performance evaluated from 3-point bending test; (A) schematic illustration of three-point bending test setup; (B) setup for the three-point bending test; (C) representative stress–deflection curve obtained from the three-point bending test of the scaffold; (D) Weibull plot of measured flexural strength; (E) overview micrograph of the tested scaffold; and (F) detailed micrograph of the crack initiation site (marked by an arrow).

The mechanical performance of prepared CaP scaffolds was evaluated from a three-point bending test (Figure 3A,B) and a compression test (S1. Compressive strength testing) showed promising results. Testing resulted in a mean flexural strength of 10.6 ± 2.7 MPa. The stress–deflection curve of one test showing a linear elastic region followed by abrupt failure typical for brittle ceramics can be seen in Figure 3C. The Weibull strength was determined to be 11.6 MPa, and the Weibull modulus was 4.4 MPa, indicating a higher scatter of data (Figure 3D) when compared with published data^31^ but still within the range for complex microstructures of CaP-based scaffolds.^32^

Considering the relative porosity of ∼70%, the flexural strength, with a value of over 10 MPa, can be deemed very good. In the case of freeze-cast scaffolds with lamellar microstructure, the mechanical properties are influenced mainly by the size of interlamellar spaces.^6,8,9^ From the application point of view, the obtained mechanical stability mainly corresponds with that of cancellous bone or articular cartilage.^33^ Relatively low Weibull modulus is attributed to the higher porosity and higher local heterogeneity in the microstructure, given by the processing technique used. The circular cross-section restricted the distribution of critical flaws in loaded volume, i.e., the loaded volume is relatively small.

Figure 3E shows that the crack initiated opposite the loading site near the specimen surface. Detailed observation (Figure 3F) showed that the lamellae (perpendicular to the fracture surface) deflected the propagating crack; hence, the fracture surface was rough. However, the microstructure formed by freeze-casting is beneficial for bending loading in the given direction, where elongated lamellae avoid interlaminar fracture. Considering the application in soft tissue, the obtained mechanical stability could be perceived as too high because the mechanical strength of human soft tissue varies in the order of units to hundreds of kPa.^15,34^ However, in the body environment, where the scaffold is in direct contact with body fluids and with increasing time since implantation, the CaP scaffolds gradually dissolve and degrade, thus losing mechanical properties.^12^ Scaffolds implanted subcutaneously for only 6 weeks showed no observable decrease in mechanical stability. In comparison, scaffolds implanted for 6 months were more fragile and easily disintegrated, although reinforced with ingrown tissue.

### 3.4 Bioactivity

**Figure 4:**
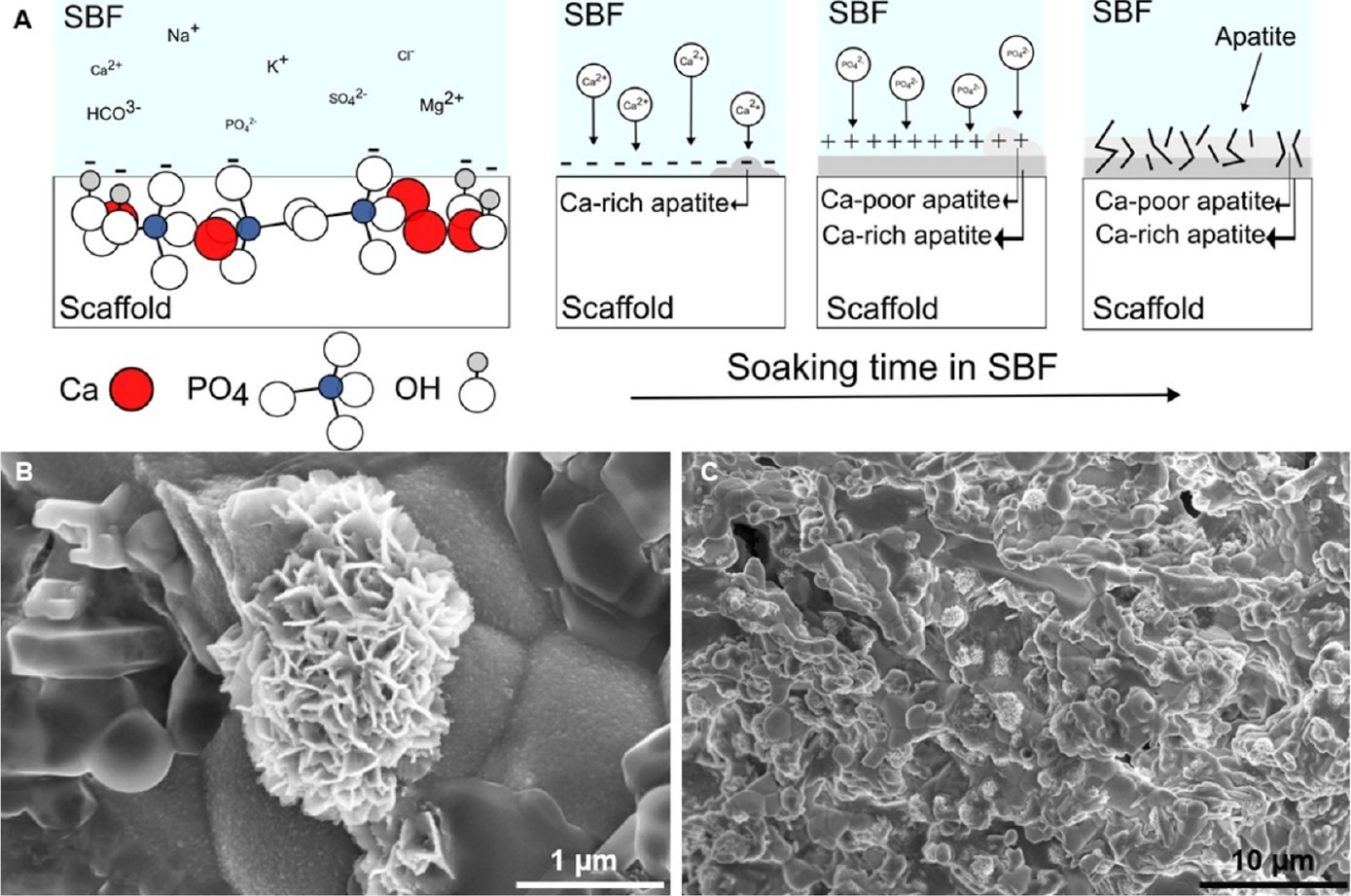
Apatite precipitated from SBF; (A) schematic illustration of apatite formation; (B) detailed view; and (C) the scaffold surface with precipitated apatite.

The SBF immersion studies are recognized as a standardized test for evaluating the material’s bioactivity. Therefore, the sintered CaP scaffolds were soaked in SBF for 21 days. The CaP-based bioceramics we tested in the SBF demonstrated their bioactivity through an increase in weight by ∼26%, indicating that the apatite layer had formed during the soaking in the SBF. Figure 4A shows the process of apatite formation on the CaP scaffold surface. Additional SEM observation proved the presence of a typical cauliflower structure of bone-like apatite (Figure 4B). The apatite did not grow homogeneously into the coherent layer but only in small regions (Figure 4C). However, this finding still leads to high bioactivity of the resulting CaP-based bioceramics, and together with porosity and pore size, will ensure protein and cell adhesion in an in vivo environment.^17,35,36^

### 3.5 Biological Evaluation

Contemplating all morphological and mechanical features of CaP-based scaffolds, the biological behavior (biocompatibility, potential toxicity, and degradation) of the prepared scaffolds was tested in vivo after subcutaneous implantation in rats. All tested groups had a regular and symptomless recovery period. After surgery, all wounds showed primary wound healing. Animals showed no distress or signs of infection, and all survived until the day of sample collection.

After scaffold retrieval, we did not observe any inflammation or pathological response in the surrounding tissue or detect any pathology of the internal organs during macropsy. Similarly, the blood counts showed no significant changes compared to the sham-operated control group (Table 1), confirming no systemic reaction to implanted scaffolds and no surgical complications, such as abscess formation.

**Table 1:** Blood Count Changes in Sham-Operated and CaP Scaffold Implanted Groups [Average Value ± Standard Deviation (SD)]

	6 weeks		6 months	
	control	CaP	control	CaP
RBC (10^6^/μL)	8.56 ± 0.32	8.48 ± 0.34	7.18 ± 0.22	7.07 ± 0.28
HCT (vol %)	0.47 ± 0.01	0.47 ± 0.01	0.48 ± 0.01	0.47 ± 0.01
PLT (10^3^/μL)	773 ± 60	755 ± 88	788 ± 110	730 ± 46
WBC (10^3^/μL)	3.30 ± 1.34	3.25 ± 1.45	2.90 ± 0.62	2.09 ± 0.68
LYM (10^3^/μL)	1.75 ± 0.68	1.76 ± 0.85	1.91 ± 0.54	1.24 ± 0.56
MONO (10^3^/μL)	0.40 ± 0.24	0.44 ± 0.26	0.38 ± 0.18	0.21 ± 0.05
NEU (10^3^/μL)	1.09 ± 0.42	0.98 ± 0.33	0.47 ± 0.18	0.51 ± 0.12
EOS (10^3^/μL)	0.02 ± 0.01	0.03 ± 0.01	0.14 ± 0.07	0.12 ± 0.03
BAS (10^3^/μL)	0.03 ± 0.02	0.03 ± 0.02	0.00 ± 0.00	0.00 ± 0.00

No significant differences were found (data distribution was tested using Kolmogorov–Smirnov and Shapiro–Wilk tests. Data with normal distribution were then assessed using the independent sample *t*-test, while non-normal values were evaluated using the Mann–Whitney test. Differences were considered significant when *p* ≤ 0.05); RBC—red blood cells, HCT—hematocrit, PLT—platelets, WBC—white blood cells, LYM—lymphocytes, MONO—monocytes, NEU—neutrophils, EOS—eosinophils, and BAS—basophils.

**Figure 5:**
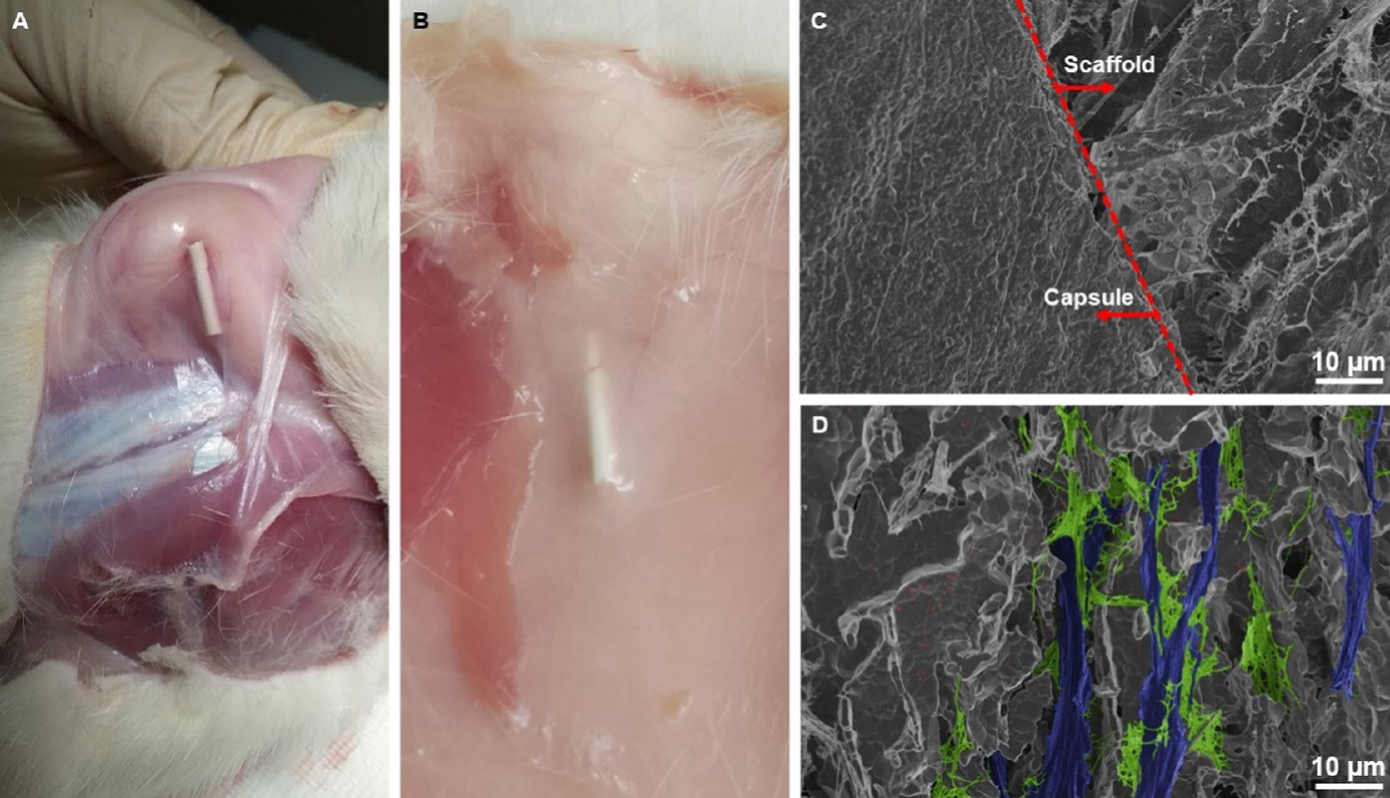
Harvested samples and their SEM observation; (A) attachment of the scaffold to skin tissue by a fibrous capsule after 6 weeks; (B) harvested sample with ingrowth blood vessels after 6 months; (C) SEM observation of capsule (left) and scaffold (right) interface; and (D) colorized SEM micrograph of tissue adhesion (fibroblastic cells–blue and collagenous fibers–green).

After subcutaneous implantation, a fine fibrous encapsulation formed around the material (Figure 5A). Macroscopically, a few vessels grew over the scaffold, and no signs of inflammation were found (Figure 5B).

SEM observation after 6 months revealed the capsule–scaffold interface and fibrous capsule reacting with the scaffold’s material (Figure 5C,D). Fibroblastic cells (blue) exposing collagenous fibers (green) were detected inside the scaffold after both implantation periods (6 weeks and 6 months). Furthermore, all potential advantages of bioceramic scaffolds, such as mechanical stability and biomimetic microstructure, were to some extent preserved in vivo, even for an extended period of 6 months. No calcification of the surrounding soft tissue or ectopic bone formation was observed due to small pores.^4,14^ Lack of calcification also signals a reasonable dissolution rate achieved by an appropriate phase composition.

**Figure 6:**
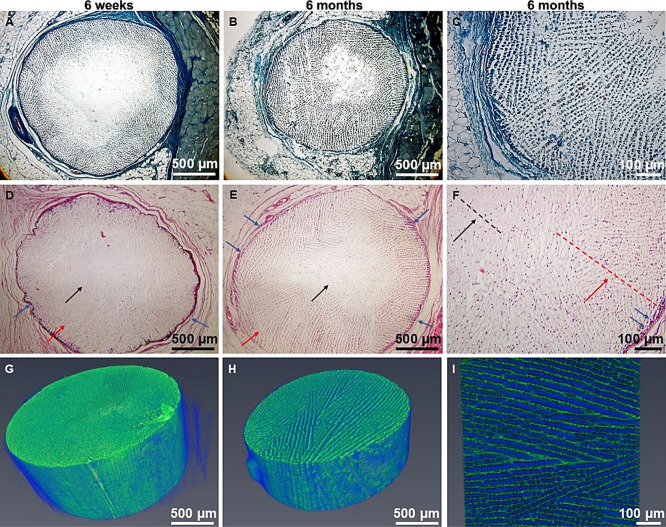
Degree of tissue ingrowth after subcutaneous implantation; (A) trichrome-stained sample; (B) trichrome-stained sample; (C) detail of fiber tissue ingrowth; (D) hematoxylin-eosin-stained sample; (E) hematoxylin-eosin-stained sample; (F) detail view on hematoxylin-eosin-stained sample (dashed lines indicate the border between the center part and side part of the scaffold); (G) CT micrograph of sample; (H) CT micrograph of sample; and (I) detail view on lamellar structure (green) and fiber tissue (blue). Blue arrows mark the outer fibrous capsule, red arrows mark ingrowing tissue and vessels, and black arrows indicate the disintegrated central part of the scaffold. In order to improve visibility, the contrast and colors were augmented.

Microscopically, three different layers could be recognized after decalcification of the CaP scaffolds (Figure 6A,B,C). The outer layer of collagen fibers (capsule, marked with blue arrows) was followed by an area with ingrowing fiber tissue and vessels (indicated by red arrows), completely disintegrating the original lamellar character (indicated by black arrows). The center displayed a lamellar arrangement (Figure 6D,E,F). The extent of disintegration increased between 6 weeks and 6 months (Table 2).

**Table 2:** Fibrous Tissue, α-SMA Positivity, Maximum Vessel Ingrowth Into CaP Scaffolds, and Macrophage Infiltration 6 Weeks and 6 Months after Subcutaneous Implantation

	6 weeks	6 months
fibrous tissue ingrowth (Masson trichrome)^a^	1.8 ± 0.4	2.9 ± 0.7^d^
αSMA positive cells^a^	1.8 ± 0.3	2.8 ± 0.7^d^
maximum vessel ingrowth (Masson Trichrome)^b^	137 ± 42	239 ± 81^d^
maximum vessel ingrowth (CD31 positive cells)^b^	127 ± 38	252 ± 92^d^
macrophages (CD68 positive cells)^c^	1.1 ± 0.6	1.2 ± 0.5

a Average value ± SD scored from the fiber capsule to the center of the samples according to the semiquantitative scale as 0 (none), 1 (1–25%), 2 (26–50%), 3 (51–75%), 4 (76–99%), and 5 (complete).

b Average value ± SD in μm measured from the fiber capsule.

c Average value ± SD scored according to the semiquantitative scale as 0 (baseline), 1 (mildly increased), 2 (moderately increased), and 3 (highly increased).

d Significantly different compared to the six-week group (*p* ≤ 0.05; see Table 1 for the statistics).

The histological results correspond with those of the CT scan (Figure 6G,H,I). The scans show notable differences between the implantation periods. The scaffolds collected after 6 weeks exhibited less tissue ingrowth (less blue color) than those collected after 6 months, which also evidenced more profound degradation of the scaffold’s lamellae (green color).

**Figure 7:**
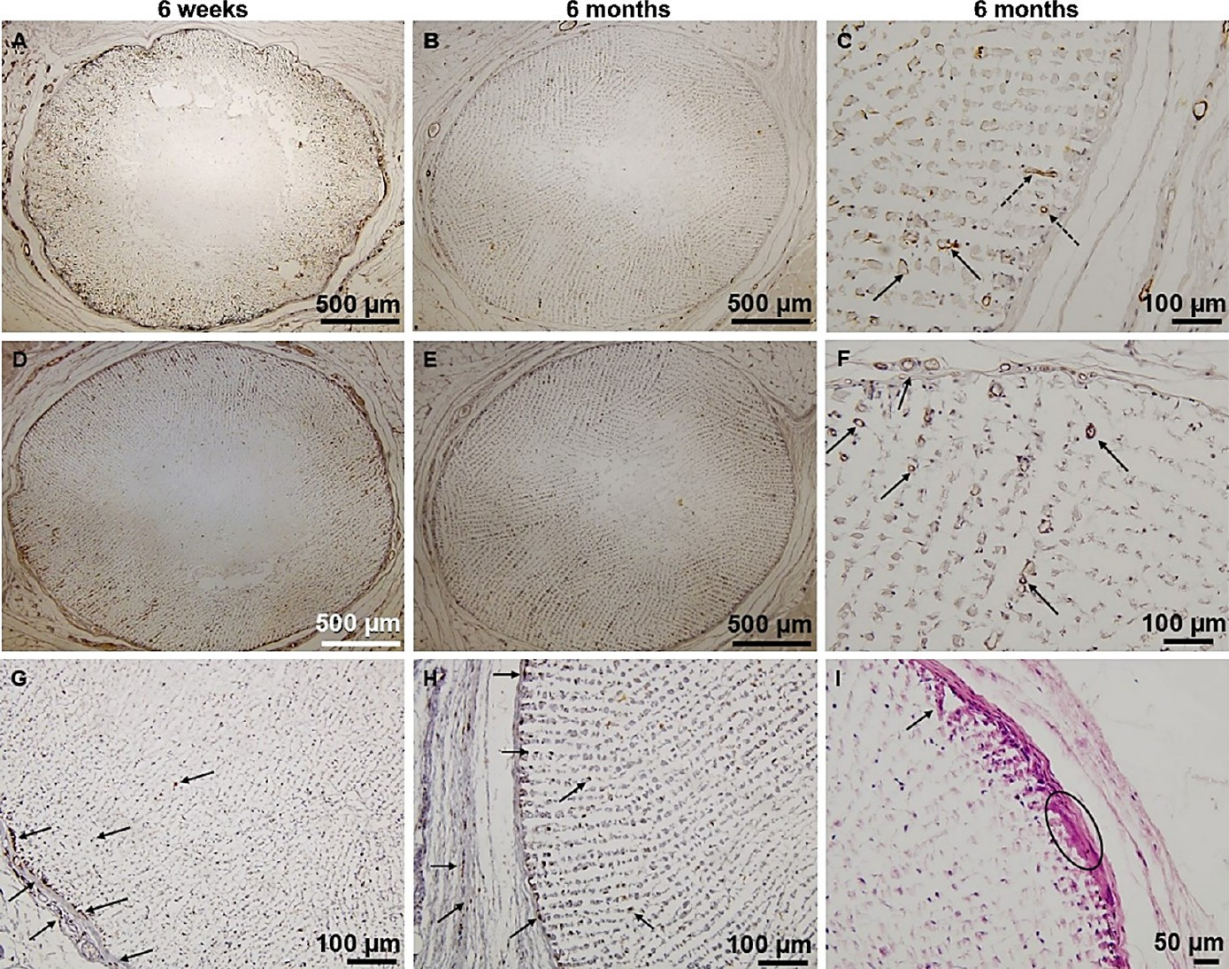
Immunohistochemistry assessment; (A) α-SMA positivity of sample; (B) α-SMA positivity of sample; (C) detail of SMA positivity in newly formed vessels (black arrows indicate α-SMA positive cells on collagen and black dashed arrows indicate αSMA positive cells on vessels); (D) CD31 positivity of sample; (E) CD31 positivity of sample; (F) detail view on CD31 positivity (black arrows mark capillaries); (G) detail view on CD68 positivity of sample (black arrows mark the macrophage infiltration); (H) detail view on CD68 positivity of sample (black arrows mark the macrophage infiltration); and (I) hematoxylin-eosin-stained sample displaying a multinucleated giant cell (MNGC) at the scaffold–capsule interface (marked with black arrow and oval). In order to improve visibility, the contrast and colors were augmented.

αSMA positivity corresponded to the fiber tissue ingrowth (Figure 7A,B,C). Most αSMA positive cells were localized at the interface of collagen fibers and disintegrated lamellae (Figure 7C, black arrow), indicating that the αSMA positive cells may be myofibroblasts.^37^ Although the marker can also detect vessels, αSMA positivity around vessel-like structures was detected only when larger structures had been formed (Figure 7C, marked with black dashed arrows).

To detect vascularization, we utilized antibodies against CD31 (Figure 7D,E,F). The vascular supply is central to most tissue-regenerative strategies. Compared with calcium sulfate or magnesium phosphate scaffolds, CaP scaffolds induced a greater volume of blood vessels.^38^ In our study, the process of vascularization and angiogenesis was successful, even though the initial pore size (size of interlamellar spaces) was low. Figure 7F shows that capillaries form around and proliferate into implanted scaffolds (marked with black arrows).

The local immune system response was primarily characterized by mildly increased macrophage (CD68 positive cells) infiltration in the outer capsule and the peripheral parts of the scaffolds (Table 2 and Figure 7G,H), with their migration exceeding the boundaries of connective tissue ingrowth toward the lamellar center (Figure 7G). In response to foreign bodies, macrophages may fuse into MNGCs.^36,37^ The biological role of MNGCs remains unknown. However, Zhao et al. suggest their formation accelerates biodegradation rates of CaP bioceramics in vivo.^39^ In our study, MNGCs were mainly present in small amounts at the scaffold–capsule interface but not in the connective tissue growing in the material (Figure 7I).

All of these findings indicate the high biocompatibility of the tested scaffolds. We did not observe any signs of toxicity, either local to the surrounding soft tissues or systemic. The response to the implanted scaffolds was mild, including encapsulation, subtle ingrowth of fibrous tissue with vessels into the material in a temporal manner, and a mild immune response, which was proportional to the slow degradation rate of the scaffolds. At the same time, the scaffolds preserved their lamellar structure during the whole testing period. This is a critical part of the study because we can assume that the designed pore size remains stable in the short term with a gradual increase over time. This precise control allows selective cell access, favoring smaller cells, while restricting others. The future perspective can potentially target nutrition or drug delivery only for cells with a specific size (function). We have also demonstrated that colonization of the whole volume is possible due to its compact design. Future research can lead toward the development of miniaturized bioreactors embedded in various soft tissues, offering a potential solution to compensate for deficiencies in the organism.

## 4 Conclusions

In this study, we have prepared highly porous CaP-based scaffolds with defined pore size and porosity via freeze-casting. The scaffolds were primarily composed of β-TCP, and the fine and oriented microstructures demonstrated robust mechanical resistance. Furthermore, the scaffolds showed high bioactivity by the apatite precipitation, which made them suitable for in vivo testing, which was conducted by the subcutaneous implantation in female rats for 6 weeks and 6 months, respectively. After each period, autopsy, blood counts, histology, and immunohistochemistry were done to determine any sign of infection, negative immune response, soft tissue calcification, tissue ingrowth, and angiogenesis. All of the aforementioned assessments showed no adverse reactions to the proposed scaffolds from an organism. Furthermore, there was no evidence of calcification in the surrounding soft tissue, which is likely to be attributed to the very narrow interlamellar spaces created by controlled freezing. Therefore, it can be concluded that such scaffolds can be considered suitable for long-term use in soft tissue regeneration. However, the authors are aware that further in vitro analyses are necessary to ascertain the precise pore size range and material composition for specific cell or tissue types.

## Supporting Information Available

Scaffold’s mechanical performance and compressive strength measurement (PDF)

## Abbreviations

HA: hydroxyapatite
TCP: tricalcium phosphate
CaP: calcium phosphate
PVA: poly(vinyl) alcohol
PLA: poly(lactic) acid
XRD: X-ray diffraction
BET: Brunauer–Emmett–Teller
SBF: simulated body fluid
SEM: scanning electron microscopy
CT: computed tomography
DPX: dibutyl phthalate polystyrene xylene
αSMA: alpha-smooth muscle actin
MNGCs: multinucleated giant cells
SD: standard deviation.
