# Supplementary material for: In Vivo Assessment on Freeze-Cast Calcium Phosphate-Based Scaffolds with a Selective Cell/Tissue Ingrowth
Source: ACS Appl Mater Interfaces. 2024 Oct 21;16(43):58326–36. doi: 10.1021/acsami.4c12715 (PMC11533149; doi:10.1021/acsami.4c12715)
Supplement: Supplementary file 1 — am4c12715_si_001.pdf [file am4c12715_si_001.pdf]

Supporting Information

In vivo assessment on freeze-cast calcium phosphate-based scaffolds with a selective cell/tissue ingrowth

Lucie Pejchalová[1], Jaroslav Pejchal[2], Jakub Roleček[1], Michaela Vojníková[1,3], Zdeněk Chlup[4], Vojtěch Mařák[1], Manuela González-Sánchez[5], Jana Čížková[6], David Salamon[1, 7]**

[1]Central European Institute of Technology, Brno University of Technology, Purkynova 656/123, 612 00 Brno, Czech Republic

[2]Department of Toxicology and Military Pharmacy Faculty of Military Health Science, University of Defence, Trebesska 1575, 500 01 Hradec Kralove, Czech Republic

[3]Department of Chemistry and Biochemistry Mendel University in Brno, trida Generala Piky 1999/5, 613 00 Brno, Czech Republic

[4]Institute of Physics of Materials, Academy of Science of the Czech Republic, Zizkova 513/22, 616 62 Brno, Czech Republic

[5]Department of Physics of Condensed Matter, Faculty of Physics, University of Seville, Av. de la Reina Mercedes, S/N, Seville 41012, Spain

[6]Department of Radiobiology, Faculty of Military Health Science, University of Defence, Trebesska 1575, 500 01 Hradec Kralove, Czech Republic

[7]Institute of Structural and Functional Ceramics, Montatuniversität Leoben, Peter Tunner Strasse 5, 8700 Leoben, Austria

S1. Compressive strength testing

Method

The compressive strength of sintered freeze-cast CaP scaffolds was determined for a minimum of 10 samples, see Figure S1 (diameter of 18.4 ± 0.2 mm and height of 20.7 ± 0.2 mm) via uniaxial compression test using a universal testing frame AG-IS (Shimadzu Autograph, Japan). The used cross-head speed was 0.6 mm/min. The compressive strength of scaffolds was calculated from the maximum stress measured during the test.

Results

Testing the sintered freeze-cast CaP scaffolds resulted in a mean compressive strength of 15.2 ± 1.9 MPa.
